# Long-COVID Prevalence and Its Association with Health Outcomes in the Post-Vaccine and Antiviral-Availability Era

**DOI:** 10.3390/jcm13051208

**Published:** 2024-02-21

**Authors:** Ramida Jangnin, Worraya Ritruangroj, Sirada Kittisupkajorn, Pattarapa Sukeiam, Juthamas Inchai, Benchalak Maneeton, Narong Maneetorn, Jindarat Chaiard, Theerakorn Theerakittikul

**Affiliations:** 1 Faculty of Medicine, Chiang Mai University, Chiang Mai 50200, Thailand; ramida_jangnin@cmu.ac.th (R.J.); worraya_rit@cmu.ac.th (W.R.); sirada_kittisup@cmu.ac.th (S.K.); pattarapa_suk@cmu.ac.th (P.S.); 2 Division of Pulmonary, Critical Care and Allergy, Department of Internal Medicine, Faculty of Medicine, Chiang Mai University, Chiang Mai 50200, Thailand; juthamas.i@cmu.ac.th; 3 Department of Psychiatry, Faculty of Medicine, Chiang Mai University, Chiang Mai 50200, Thailand; benchalak.maneeton@cmu.ac.th (B.M.); narong.m@cmu.ac.th (N.M.); 4 Faculty of Nursing, Chiang Mai University, Chiang Mai 50200, Thailand; jindarat.c@cmu.ac.th; 5 Sleep Disorder Center, Center for Medical Excellence, Faculty of Medicine, Chiang Mai University, Chiang Mai 50200, Thailand

**Keywords:** long COVID, COVID-19, health impact, post-vaccinated, quality of life

## Abstract

**Background and Objectives:** After recovering from COVID-19, patients may experience persistent symptoms, known as post-COVID-19 syndrome or long COVID, which include a range of continuing health problems. This research explores the prevalence, associated factors, and overall health outcomes of long COVID during a period of extensive vaccination and antiviral treatment availability in Thailand. **Materials and Methods:** This observational study involved 390 adult patients with COVID-19 between January and March 2022. Beginning three months after their diagnosis, these patients were interviewed via telephone every three months for a period of one year. The data collection process included gathering demographic information and administering a standardized questionnaire that addressed the patients’ physical condition following COVID-19, their mental health, sleep disturbances, and overall quality of life. **Results:** The cohort consisted of 390 participants, with an average age of 31.8 ± 13.6. Among them, 96.7% (*n* = 377) were vaccinated, and 98.2% (*n* = 383) underwent antiviral treatment. Long-COVID prevalence was observed at 77.7%, with the most frequently reported symptoms being fatigue (64.1%) and cough (43.9%). Regarding mental health, depression was reported by 8.2% of the participants, anxiety by 4.1%, and poor sleep quality by 33.3%. Advanced statistical analysis using multivariable logistic regression showed significant links between long-COVID symptoms and patients aged below 60 (*p* = 0.042), as well as the initial symptom of cough (*p* = 0.045). In the subset of long-COVID sufferers, there was a notable correlation in females with symptoms such as headaches (*p* = 0.001), dizziness (*p* = 0.007), and brain fog (*p* = 0.013). **Conclusions:** Despite the extensive distribution of vaccines and antiviral therapies, the prevalence of long COVID remains high, being associated particularly with individuals under 60 and those exhibiting a cough as an early symptom. The study further reveals that mental health issues related to long COVID are profound, going beyond the scope of physical symptomatology.

## 1. Introduction

The emergence of the severe acute respiratory syndrome coronavirus 2 (SARS-CoV-2), causing the ongoing COVID-19 pandemic, has significantly impacted global health since 2019. The spectrum of clinical presentations among COVID-19 patients range from asymptomatic to severe pneumonia [1], potentially progressing to life-threatening conditions, especially in individuals with comorbidities. SARS-CoV-2 utilizes host cells with ACE-2 receptors, leading to inflammation in various systems such as the cardiovascular, gastrointestinal, nervous, and urinary systems, resulting in multiple-organ-dysfunction syndrome (MODS) [2,3].

As of 17 May 2021, the Department of Disease Control, Ministry of Public Health of Thailand, reported an accumulation of 255,341,861 global COVID-19 cases and 2,037,224 cases within Thailand [4]. Approximately 90% of recovered patients report developing chronic symptoms or ‘Long COVID’, according to research by Marwa Kamal et al. [5].

The World Health Organization defines ‘Long COVID’ or ‘Post COVID-19 syndrome’ as the persistence or development of symptoms beyond 12 weeks in individuals with a history of probable or confirmed SARS-CoV-2 infection [6]. Typically commencing three months post-onset of COVID-19, these symptoms endure for at least two months and cannot be attributed to an alternate diagnosis. Presentations of long COVID vary, with commonly reported symptoms including fatigue, shortness of breath, and headache [7,8]. More severe symptoms may involve renal failure, pulmonary fibrosis, myocarditis, arrhythmia [5], and others. This wide range of symptoms made the reported prevalence varied between populations across the globe. The cross sectional study from a community setting in a southern part of Thailand with undetermined vaccination status report the long-COVID prevalence to be as high as 79.3% [9].

Reports also indicate cognitive and psychological effects such as attention deficit, sleep disturbance, depression, and post-traumatic stress disorder [10]. Age, comorbidity, and severity of initial COVID-19 infection have been associated with long-COVID presentations [11].

Amid the fifth wave of the COVID-19 pandemic in Thailand, marked by the dominance of the Omicron variant since January 2022 [12], there has been a significant increase in national vaccination coverage and antiviral drug use. Despite the decrease in severe and mechanically ventilated COVID-19 cases during this wave, there remains a dearth of information on long COVID during this period of the Omicron variant’s dominance and post-vaccination.

Previous studies emphasize that physical, mental, and social factors significantly contribute to health and disease incidence, underscoring the importance of social support in patient recovery [13,14]. As such, our study aims to explore the prevalence of long COVID, associated factors, and holistic outcomes in the era of widespread vaccination and antiviral availability in Thailand.

## 2. Method

### 2.1. Study Design

The Research Ethics Committee, Faculty of Medicine, Chiang Mai University, approved this prospective observational study (Study code: MED-2564-08702, date of approval: 3 February 2022). We confirm that this research was performed in accordance with the relevant Declaration of Helsinki guidelines/regulations. Informed consent was acquired from all participants. This study was conducted on subjects with positive COVID-19 Antigen Test Kits (ATK) or reverse transcription-Polymerase Chain Reaction (RT-PCR) tests from January–April 2022 in Maharaj Nakorn Chiang Mai Hospital—Faculty of Medicine, Chiang Mai University. Subjects who met the inclusion criteria were invited to participate. Sample size was determined using the applied Taro Yamane Formula, allowing a 5% margin of error to reflect the population affected by long COVID. In total, 390 subjects who committed with informed consent were traced back to their medical records of symptoms at their positive test date and interviewed telephonically to collect information about long-COVID-19 manifestations. The phone interviews started three months after the day of diagnosis and continued every quarter for a year. The questionnaire was divided into four parts: demographic data, post-COVID physical manifestations, post-COVID mental effects, and sleep effects.

### 2.2. Data Collection

We collected demographic data, including age, gender, BMI, comorbidities, receipt of COVID vaccine, length of stay, receipt of antiviral drugs, and receipt of oxygen therapy. The interview questionnaires contained four aspects of the standard scale including post-COVID-19 information on physical conditions, post-COVID mental effects [depression: patient health questionnaire (PHQ-9) [15], anxiety: general anxiety disorder-7 (GAD-7), post-traumatic stress disease: the screening questionnaire for PTSD (2P) and the psychological impact scale for crisis events (PISCES-10) [16]], sleep effects [sleep quality: Thai Version of the Pittsburgh Sleep Quality Index (Thai-PSQI) [17], daytime sleepiness: Thai Version of Epworth sleepiness scale (Thai-ESS [17]), daytime function: Functional Outcomes of Sleep questionnaire (FOSQ-10) [17]] and quality of life after COVID-19 recovery [12-item short-form survey (SF-12) [18]].

All participants were asked about post-COVID physical manifestations based on their types of symptoms, including onset and duration of symptoms, general symptoms (fatigue, fever, chill), respiratory symptoms (dyspnea, cough), cardiovascular symptoms (palpitation, tachycardia, chest pain), neurological symptoms (anosmia, headache, dizziness, abnormal movements, brain fog syndrome, attention deficit), gastrointestinal symptoms (diarrhea, nausea and vomiting, abdominal pain), skin (rash, alopecia, desquamation), ear—nose—throat symptoms (dysphagia, hearing loss, blurred vision, ear pain), musculoskeletal symptoms (myalgia, bone and joint pain), immunological symptoms (worsening hypersensitivity, new-onset hypersensitivity, herpetiform dermatitis), and reproductive symptoms (testicular pain, impotence).

The PHQ-9 questionnaire [15] measures levels of depression and contains nine items. Each item has a score of 0–3, giving the maximum score of 27. The total score of 5, 10, 15, and 20 represent mild, moderate, moderately severe, and severe depression, respectively.

The GAD-7 questionnaire measures levels of generalized anxiety disorder and contains seven items. Each item has a score of 0–3, giving the maximum score of 21, and a score of 8 or greater represents a cut point for identifying generalized anxiety disorder. Anxiety severity levels correlate with a score of 0–4: minimal anxiety, 5–9: mild anxiety, 10–14: moderate anxiety, and more than 15: severe anxiety.

The 2P questionnaire [16] for primary screening of post-traumatic stress disorder contains two yes/no questions. Suppose the participants answer yes to both questions, it indicates a risk of PTSD. The participants will take PISCES-10, which measures the physiological impact scale for crisis events. The PISCES-10 [16] contains ten items. Each item has a score of 0–3, giving the maximum score of 30. The score range 0–8 represents minimal or no risk of PTSD, 9–18 describes a moderate risk of PTSD, and a score of more than 19 is considered a high risk of PTSD [16].

The Pittsburgh Sleep Quality Index (Thai-PSQI) [17] is an instrument for assessing sleep quality and behavior. It contains 19 items that can be grouped to determine seven aspects: subjective sleep quality, sleep latency, sleep duration, habitual sleep efficiency, sleep disturbance, use of sleep medication, and daytime dysfunction. If the summation of seven subscales exceeds five, its considered poor sleep quality.

The Epworth Sleepiness Scale (Thai-ESS) [17] assesses daytime sleepiness and contains eight questions. Each question is a regular activity with a 0–3, and a total score ranges from 0–24. An ESS score of more than 10 represents excessive daytime sleepiness, a score of 11–14 is considered mild sleepiness, a score of 15–17 is regarded as moderate sleepiness, and a score of 18–24 is considered severe sleepiness.

The Functional Outcomes of Sleep Questionnaire (FOSQ-10) [17] contains ten questions that evaluate the impact of daytime sleepiness on activities in daily life. Each question has five scales ranging from 0 to 4, with a maximum score of 40. The higher scores on the FOSQ indicate better functional outcomes, while a lower score on the FOSQ indicates worse daytime quality, as it suggests that the individual is experiencing difficulties with their daily functioning due to poor sleep quality.

**Figure 1:**
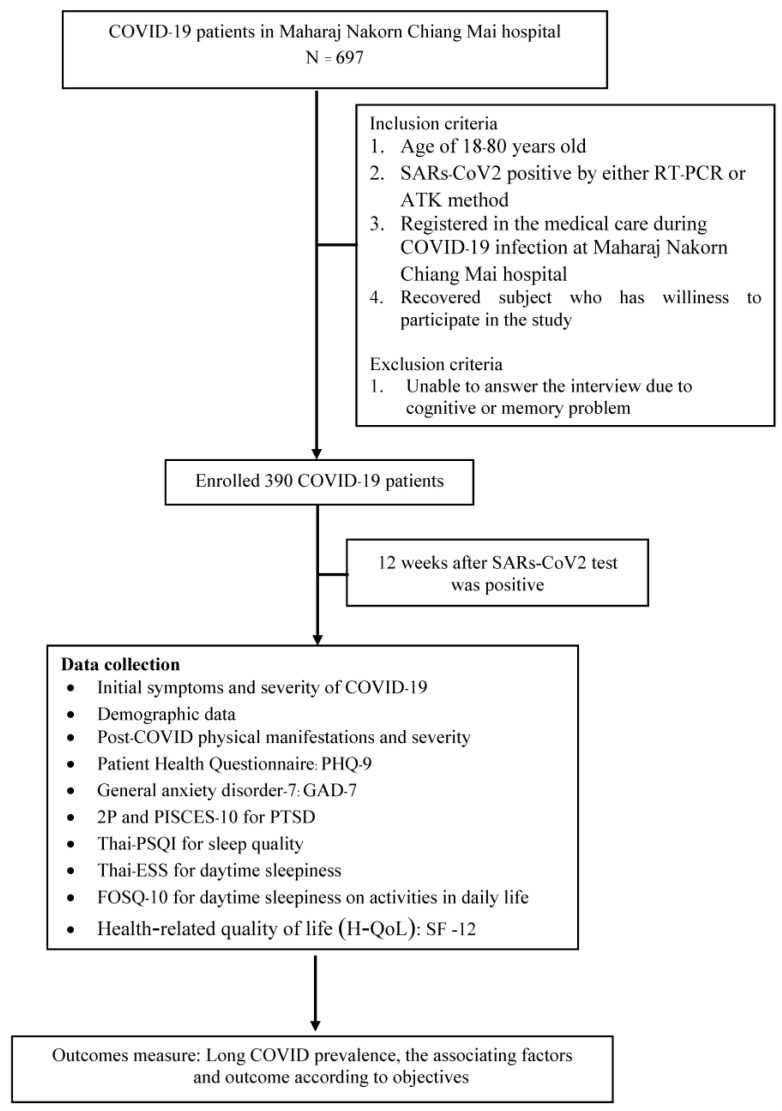
Flow chart of study.

The SF-12 [18], a short version of the SF-36, is used to evaluate health-related quality of life (H-QoL). This questionnaire consists of 12 items divided into two sub-dimensions which are physical and psychosocial health. The total score ranging from 12 to 47 was transformed into a scale from 0 to 100, with higher scores indicating better quality of life. The recommended cut-off score to determine the poor quality of life is 50 or less. The study flow as shown in Figure 1.

### 2.3. Statistical Analysis

Categorical variables were denoted as counts and percentages, while continuous variables were expressed as means with standard deviation (S.D.) or medians with interquartile range (IQR), contingent on the distribution normality as verified by the Kolmogorov–Smirnov test. Fisher’s exact test was employed to compare demographic and clinical variables between long-COVID and non-long-COVID patients for categorical data, while Student’s *t*-test or the Wilcoxon rank sum test was used for continuous variables, depending on the data distribution. Logistic regression served to identify independent factors associated with long COVID. Variables with a *p*-value ≤ 0.20 from univariable analysis and those with clinical relevance were included in the multivariable analysis to ascertain independent associated factors. The results were provided as adjusted odds ratios (OR) and 95% confidence intervals (CI), considering *p* < 0.05 as statistically significant. All statistical analyses were conducted using the STATA Statistical Package, version 14.0.

## 3. Results

There were 390 patients with a mean age of 31.8 ± 13.6. The study consisted of 55.6% female participants and 44% male participants. Out of the total number of patients, 377 (96.7%) received vaccinations, while 383 (98.2%) were administered antiviral drugs, respectively. The majority of participants in the study (94.8%) received at least two doses of the COVID-19 vaccine, with 40.5% receiving two doses and 54.3% receiving a booster dose. Among the participants, 32.6% were students and 27.7% were healthcare workers. Most of the participants had a normal BMI and no comorbidities. During the course of the disease, 96.4% of participants did not require oxygen therapy. The most commonly reported initial symptoms of COVID-19 infection were cough (44.9%), sore throat (35%), and rhinorrhea (18.2%) as shown in Table 1.

**Figure 2:**
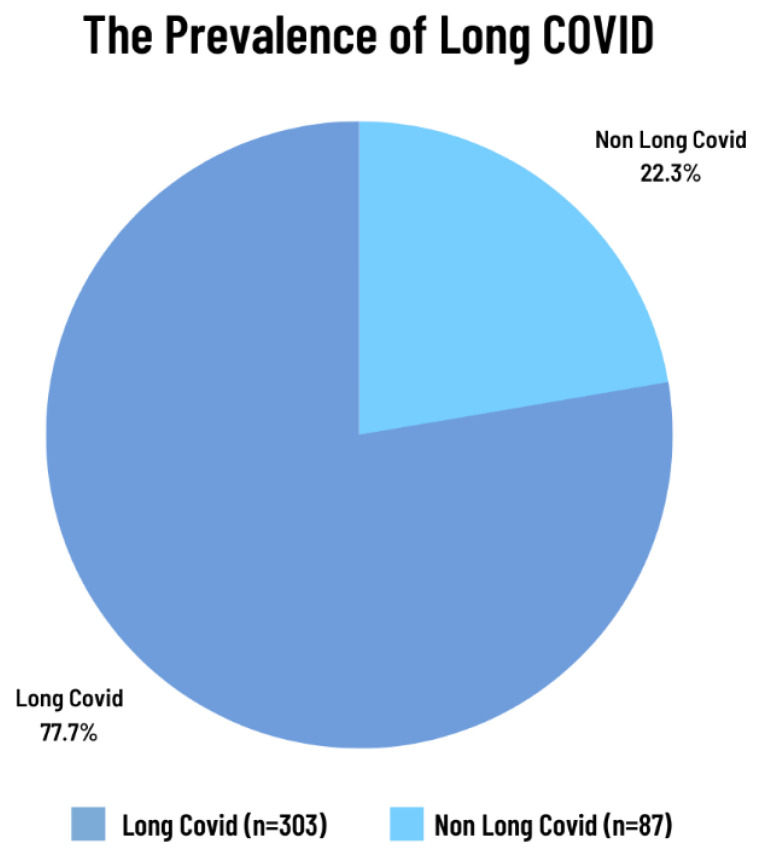
The prevalence of long COVID.

**Figure 3:**
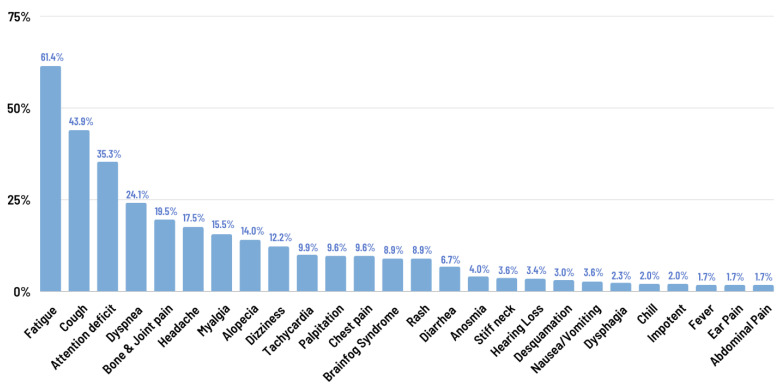
Long-COVID symptoms.

The prevalence of long COVID was found to be 77.7% (Figure 2), with the most commonly reported residual symptoms being fatigue (64.1%) and cough (43.9%) (Figure 3). Mental health was assessed in terms of depression, anxiety, and post-traumatic stress disorder (PTSD) (Table 2). Overall, 91.8% of patients had no or minimal depression, while 8.2% of patients experienced mild depression (4.4%), moderate depression (3.3%), or moderately severe depression (0.5%). Most patients did not experience significant anxiety after the remission of COVID-19, with only 4.1% of patients experiencing mild to moderate anxiety.

Poor sleep quality was reported in 33.3% of patients, and it was significantly higher in the long COVID group (38.1% vs. 16.5%, *p* < 0.001). Additionally, the health-related quality of life (H-QoL:SF-12) was significantly lower in the long COVID group even after the remission of COVID-19 infection, as shown in Table 2.

Univariable and multivariable logistic regression analyses were performed to identify factors associated with long-COVID symptoms (Table 3). The analysis revealed that an age of less than 60 (*p* = 0.042) and having a cough as an initial symptom (*p* = 0.045) were significantly associated with long-COVID symptoms. When analyzing the data by gender, it was found that female patients were significantly more likely to experience headaches (*p* = 0.001), dizziness (*p* = 0.007), and brain fog syndrome (*p* = 0.013) (Table 4).

## 4. Discussion

In this prospective observational study, we aimed to determine the prevalence of long COVID and its associated factors among patients during the fifth wave of the COVID-19 pandemic, dominated by the Omicron variant. We observed a decrease in severe and mechanically ventilated COVID-19 cases compared to the previous waves (dominated by Alpha and Delta variants). This decline correlates with increased national COVID-19 vaccination coverage, multidose boosting vaccinations, and the accessibility of antiviral drugs. Most patients had received both vaccinations and antiviral drugs. The most frequently reported initial symptoms during this wave were a cough and sore throat.

Among 390 patients, 77.7% exhibited long-COVID symptoms, with fatigue (64.1%) and cough (43.9%) being the most common physical symptoms. Significantly, the presence of a cough was correlated with long-COVID development (*p*-value 0.045), possibly due to the persistent viral activity, immune dysregulation, and tissue damage.

Furthermore, mental health effects were reported, with 8.2% of patients experiencing mild to moderately severe depression and 4.1% demonstrating mild to moderate anxiety post-recovery. Poor sleep quality and daytime sleepiness were reported in 33.3% and 20% of patients, respectively. We hypothesize that various biopsychosocial factors contribute to these non-physical symptoms of long COVID, including stress related to confronting a potentially life-threatening illness, the physical symptoms of long COVID (such as fatigue, brain fog, and poor sleep quality), systemic inflammatory response induced by long COVID, and financial problems arising from work-related adjustments due to long COVID. Further controlled studies are encouraged to verify these hypotheses.

While a higher age has been associated with increased severity of COVID-19 and a more prolonged persistence of post-COVID symptoms [19], our logistic regression analysis identified a significant association between individuals under 60 years of age and long COVID (*p*-value 0.042). This finding diverges from the prior research and may be attributed to the Omicron variant’s heightened infectivity among younger individuals with more active social lives [20].

Previous metanalysis [19] found that, overall, female sex was significantly associated with post-COVID conditions, but subsequent analyses indicated this might result from the heterogeneity of the study populations included and may not be consistent across all studies. Interestingly, our study, which had an equal gender distribution, found a higher prevalence of certain long COVID symptoms, such as headaches (*p*-value = 0.001), dizziness (*p*-value = 0.007), and brain fog syndrome (*p*-value = 0.013) in female patients. This could suggest a greater risk of long COVID in women, potentially due to hormonal, immunological, and lifestyle differences. Future research is recommended to explore these potential factors.

Contrary to expectations, no significant link was found between the presence of comorbidities or the severity of the initial COVID-19 infection and the emergence of long-COVID symptoms. The manifestations of long COVID varied with the dominant viral variant, vaccination status, and initial symptoms during different pandemic waves [8,21]. Our findings indicate that the Omicron variant, alongside widespread vaccination and antiviral treatment availability, led to milder cases and a distinct symptomatology of long COVID compared to previous pandemic phases.

However, given the observational nature of our study, further prospective research is needed to establish causal relationships, and longitudinal studies are advised to track the evolution of long-COVID symptoms over time. Comparative analyses in different demographic settings or regions will aid in contextualizing the results on a global scale. Despite its limitations, our study provides valuable insight into the manifestation of long COVID during the era of vaccination and antiviral treatment availability. Our methodology, involving telephone-based interviews conducted by a single well-trained interviewer, yielded more reliable data than paper-based questionnaires. Nonetheless, we acknowledge that a limitation of these patient-reported outcomes are that they are not verifiable through face-to-face clinical evaluations due to COVID-19 restrictions at the time. As this was an observational study, the identified causes, factors, onset, duration of symptoms, and types of symptoms of long COVID were based on universally standardized questionnaires to meet the diagnostic criteria for long COVID in each patient. Consequently, further causative research is suggested to develop specific parameters for this unique population.

## 5. Conclusions

Our study identified a significant prevalence of long COVID during a period characterized by extensive vaccination and antiviral treatment availability. Notably, there was a significant relationship between long COVID and factors such as age and the occurrence of a cough. Among the symptoms of long COVID, fatigue and cough were the most commonly reported. Additionally, our findings indicated significant gender differences in the development of some long-COVID symptoms. Beyond the physical manifestations, mental health issues, notably poor sleep quality, were significantly affected. The presentation of long-COVID symptoms varied with the prevailing COVID-19 variant, levels of vaccination, and the initial symptoms, distinguishing our findings from those of studies in different settings. Contrary to expectations, the severity of the initial COVID-19 infection did not show a significant correlation with the onset of long COVID in our research. This analysis primarily focuses on the initial post-recovery phase. A comprehensive long-term evaluation is currently underway, aiming to delve deeper into the investigation of longitudinal changes in symptom severity, identify potential predictors of symptom progression, and assess the impact of various interventions on the trajectory of long COVID.

## Figures and Tables

**Table 1:** Baseline characteristics of COVID patients (*n* = 390).

Variable	Total	Long COVID (*n* = 390)		*p*-Value
		Yes (*n* = 303)	No (*n* = 87)	
Age, (y) Mean ± SD	31.8 ± 13.6	31.7 ± 14.8	31.8 ± 13.2	0.431
Male gender, N (%)	173 (44.4)	128 (42.2)	45 (51.7)	0.074
BMI, N (%)				0.281
Underweight <18.5	43 (11.0)	29 (9.6)	14 (16.1)	
Normal (18.5–24.9)	233 (59.7)	181 (59.7)	52 (59.8)	
Overweight (25–29.9)	76 (19.5)	61 (20.1)	15 (17.2)	
Obesity (>30)	38 (9.7)	32 (10.6)	6 (6.9)	
Co-morbidities, N (%)	159 (40.8)			
Obesity (>30)	38 (9.7)	32 (10.6)	6 (6.9)	0.212
Allergic rhinitis	34 (8.7)	28 (9.2)	6 (6.9)	0.33
Hypertension	33 (8.5)	27 (8.9)	6 (6.9)	0.365
Dyslipidemia	27 (6.9)	23 (7.6)	4 (4.6)	0.239
Cerebrovascular disease	16 (4.1)	11 (3.6)	5 (5.7)	0.273
Diabetes mellitus	12 (3.1)	11 (3.6)	1 (1.1)	0.211
Respirator disease	12 (3.1)	8 (2.6)	4 (4.6)	0.268
Chronic kidney disease	10 (2.6)	7 (2.3)	3 (3.4)	0.393
Gastrointestinal disease	4 (1)	4 (1.3)	0	0.363
Major depressive disorder	4 (1)	4 (1.3)	0	0.363
History of COVID-19 Vaccination, N (%)	377 (96.7)	295 (97.4)	82 (94.3)	0.14
1 shot	7 (1.8)			
2 shots	158 (40.5)			
3 shots	116 (29.7)			
4 shots	96 (24.6)			
Initial symptom during COVID-19 infection, N (%)				
Cough	175 (44.9)	144 (47.5)	31 (36.5)	0.049
Sore throat	135 (34.6)	106 (35)	29 (33.3)	0.766
Rhinorrhea	69 (17.7)	55 (18.2)	14 (16.1)	0.657
Fever	65 (16.7)	48 (15.8)	17 (19.5)	0.253
Sputum	40 (10.3)	30 (9.9)	10 (11.5)	0.666
Fatigue	13 (3.3)	10 (3.3)	3 (3.4)	0.584
Headache	11 (2.8)	9 (3)	2 (2.3)	0.739
Chest tight	9 (2.3)	9 (3)	0	0.217
Anosmia	7 (1.8)	6 (2)	1 (1.1)	0.607
Myalgia	6 (1.5)	5 (1.7)	1 (1.1)	0.738
Pneumonia	21 (5.4)	19 (6.3)	2 (2.3)	0.185
Nasal congestion	5 (1.3)	3 (1)	2 (2.3)	0.310
Receiving anti-viral drug during disease, N (%)	383 (98.2)	297 (98.3)	86 (98.9)	0.597
Receiving O_2_ therapy during disease, N (%)	14 (3.6)			0.804
Nasal canular	11 (2.8)	9 (3)	2 (2.3)	
Non invasive	2 (0.5)	2 (0.7)	0	
Mechanical ventilator	1 (0.3)	1 (0.3)	0	

**Table 2:** The impact of long COVID on mental health and quality of life.

Mental Symptoms: Parameters	Total (*n* = 390) *n* (%)	Long COVID		*p*-Value
		Yes	No	
**Depression: PHQ-9**				
None to minimal (0–4)	357 (91.8)	270 (89.1)	87 (100)	0.035
Mild (5–9)	17 (4.4)	17 (5.6)	0	
Moderate (10–14)	13 (3.3)	13 (4.3)	0	
Moderately severe (15–19)	2 (0.5)	2 (0.7)	0	
**Anxiety: GAD-7**				
No anxiety	257 (65.9)	182 (60.1)	75 (86.2)	<0.001
Minimal (1–4)	117 (30.0)	105 (34.7)	12 (13.8)	
Mild (5–9)	11 (2.8)	11 (3.6)	0	
Moderate (10–14)	5 (1.3)	5 (1.7)	0	
Severe (≥15)	0	0	0	
**PTSD: 2P, PISCES-10**				
No	3 (0.8)	3 (0.8)	0	
Yes	1 (0.3)	1 (0.3)	0	
**Sleep quality: Thai-PSQI**				
Normal	258 (66.7)	187 (61.9)	71 (83.5)	<0.001
Poor	129 (33.3)	115 (38.1)	14 (16.5)	
**Daytime sleepiness: Thai-ESS**				
Lower normal daytime symptoms (0–5)	316 (81)	242.0 (79.9)	74 (85.1)	0.390
Higher normal daytime symptoms (6–10)	65 (16.7)	52.0 (17.2)	13 (14.9)	
Mild excessive daytime symptoms (11–12)	6 (1.5)	6.0 (2)	0	
Moderate excessive daytime symptoms (13–15)	-	0	0	
Severe excessive daytime symptoms (16–24)	3 (0.8)	3.0(1.0)	0	
**Daytime function: FOSQ-10**				
Normal	387 (99.2)	300 (99.0)	87 (100)	0.351
Poor (<18)	3 (0.8)	3.0 (1.0)	0	
**H-QoL: SF-12 (Mean ± SD)**	49.0 ± 4.0			
Physical	83.25 ± 11.75	81.38 ± 12.37	89.77 ± 5.65	<0.001
Mental	82.30 ± 8.50	81.30 ± 8.87	85.78 ± 5.92	<0.001

**Table 3:** The factors associated with long-COVID symptoms using univariable and multivariable logistic regression analyses.

	Univariable Logistic Regression			Multivariable Logistic Regression		
Variable	OR	95% CI	*p*-Value	OR	95% CI	*p*-Value
**Sex**	1.465	0.908–2.363	0.118	1.431	0.874–2.344	0.154
**Age (y)**						
<60	Ref.			Ref.		
≥60	0.471	0.180–1.236	0.126	0.299	0.094–0.955	0.042
**Comorbidity**						
**Participant with ≥ 1 disease**	1.605	0.969–2.656	0.066	1.635	0.957–2.794	0.072
1. Hypertension	1.321	0.527–3.309	0.553			
2. Diabetes Mellitus	3.24	0.412–25.449	0.264			
3. Dyslipidemia	1.704	0.573–5.068	0.337			
4. Allergic rhinitis	1.375	0.55–3.435	0.496			
5. Anemia	1.151	0.127–10.429	0.901			
6. Hyperthyroid	1.761	0.209–14.828	0.603			
7. Chronic kidney disease	0.662	0.168–2.616	0.557			
8. Respiratory	0.563	0.165–1.915	0.357			
9. Gout	0.425	0.070–2.585	0.353			
10. Cerebrovascular disease	0.618	0.209–1.829	0.384			
11. Obesity	1.594	0.644–3.947	0.313			
**COVID symptoms**						
** Symptomatic COVID ≥ 1 symptom**	1.318	0.802–2.164	0.213	0.737	0.385–1.412	0.359
Cough	1.636	0.999–2.679	0.05	1.911	1.015–3.598	0.045
Fever	0.775	0.420–1.431	0.415			
Sore throat	1.076	0.650–1.782	0.776			
Sputum	0.846	0.396–1.808	0.666			
Rhinorrhea	1.156	0.608–2.198	0.657			
Anosmia	1.737	0.206–14.628	0.611			
Fatigue	0.956	0.257–3.552	0.946			
Headache	1.301	0.276–6.136	0.739			
Myalgia	1.443	0.166–12.517	0.739			
Nasal congestion	0.425	0.070–2.585	0.353			
Pneumonia	2.843	0.650–12.453	0.166	2.044	0.528–7.907	0.065

**Table 4:** The factor associated with long COVID, comparing between gender group.

Physical Symptoms	Long COVID (*n* = 303), *n* (%)	Female (*n* = 175), *n* (%)	Male (*n* = 128), *n* (%)	OR	95% CI	*p*-Value
Fatigue	186 (61.4)	110 (62.9)	76 (59.4)			
Cough	133 (43.9)	81 (46.3)	52 (40.6)			
Dyspnea	73 (24.1)	42 (24.0)	31 (24.2)			
Headache	53 (17.5)	41 (23.4)	12 (9.4)	0.338	(0.170, 0.674)	0.001
Dizziness	37 (12.2)	29 (16.6)	8 (6.3)	0.336	(0.148, 0.761)	0.007
Brain fog syndrome	27 (8.9)	22 (12.6)	5 (3.9)	0.283	(0.104, 0.768)	0.013

## Data Availability

The datasets generated and analyzed during the current study are not publicly available due to the informed consent given by COVID patients in this study which does not cover data posting in public databases. However, data are available upon reasonable request should be sent to theerakorn.t@cmu.ac.th.
